# Association between blood pressure and endovascular treatment outcomes differs by baseline perfusion and reperfusion status

**DOI:** 10.1038/s41598-023-40572-0

**Published:** 2023-08-23

**Authors:** Beom Joon Kim, Nishita Singh, Hyeran Kim, Bijoy K. Menon, Mohammed Almekhlafi, Wi-Sun Ryu, Joon-Tae Kim, Jihoon Kang, Sung Hyun Baik, Jun Yup Kim, Keon-Joo Lee, Cheolkyu Jung, Moon-Ku Han, Hee-Joon Bae

**Affiliations:** 1 Department of Neurology, Seoul National University Bundang Hospital, Office #8710, 82 Gumi-ro 173 beon-gil, Bundang-gu, Seongnam-si, Gyeonggi-do 13620 South Korea; 2 Cerebrovascular Center, Gyeonggi Regional Cardiocerebrovascular Center, Seoul National University Bundang Hospital, Seongnam-si, Gyeonggi-do South Korea; 3 Department of Neurology, Seoul National University College of Medicine, Seoul, South Korea; 4 Neurology division, Rady Faculty of Health Sciences, University of Manitoba, Winnipeg, Canada; 5 Calgary Stroke Program, Department of Clinical Neuroscience, Radiology and Community Health Sciences, University of Calgary, Calgary, AB Canada; 6 Department of Neurology, Dongguk University Ilsan Hospital, Goyang-si, South Korea; 7 Department of Neurology, Chonnam National University Medical School, Chonnam National University Hospital, Gwangju, South Korea; 8 Department of Radiology, Seoul National University Bundang Hospital, Seongnam-si, Gyeonggi-do South Korea; 9 Department of Neurology, Korea University Guro Hospital, Seoul, South Korea

**Keywords:** Stroke, Hypertension

## Abstract

We hypothesized that the association between BP and endovascular treatment (EVT) outcomes would differ by baseline perfusion and recanalization status. We identified 388 ICA or M1 occlusion patients who underwent EVT ≤ 24 h from onset with successful recanalization (TICI ≥ 2b). BP was measured at 5-min intervals from arrival and during the procedure. Systolic BPs (SBP) were summarized as dropmax (the maximal decrease over two consecutive measurements), incmax (the maximal increase), mean, coefficient of variation (cv), and standard deviation. Adequate baseline perfusion was defined as hypoperfusion intensity ratio (HIR) ≤ 0.5; infarct proportion as the volume ratio of final infarcts within the T_max_ > 6 s region. In the adequate perfusion group, infarct proportion was closely associated with SBP_dropmax_ (β ± SE (*P*-value); 1.22 ± 0.48, (< 0.01)), SBP_incmax_ (1.12 ± 0.33, (< 0.01)), SBP_cv_ (0.61 ± 0.15 (< 0.01)), SBP_sd_ (0.66 ± 0.08 (< 0.01)), and SBP_mean_ (0.71 ± 0.37 (0.053) before recanalization. The associations remained significant only in SBP_dropmax_, SBP_incmax_, and SBP_mean_ after recanalization. SBP_incmax_, SBP_cv_ and SBP_sd_ showed significant associations with modified Rankin Scale score at 3 months in the pre-recanalization period. In the poor perfusion group, none of the SBP indices was associated with any stroke outcomes regardless of recanalization status. BP may show differential associations with stroke outcomes by the recanalization and baseline perfusion status.

## Introduction

Revascularization therapy can bring a straightforward functional recovery in patients with acute ischemic stroke due to large vessel occlusion (LVO) with a number needed to treat (NNT) of 4. However, there remains a substantial proportion (54%) of patients in whom functional recovery is insufficient despite endovascular treatment (EVT)^1^. In this subset of patients, periprocedural medical treatment might be effective in achieving good functional outcomes^2^. As there are individual differences in the viability of salvageable ischemic tissue and ischemic progression after LVO^3^, modulating systemic blood pressure (BP) peri-procedurally may be a treatment target to maintain the tenacity of the ischemic penumbra before recanalization and to minimize hemorrhagic complications after EVT.

During the peri-EVT period, high or low BP measurements are usually negatively associated with the treatment outcome^4–7^. However, studies analyzing the relationship between BP level and stroke outcomes have a major limitation, as these studies do not exclude the possibility that higher BP can occur as a consequence of severe ischemia^8^. A recent randomized controlled trial (BP-TARGET) failed to show a benefit in functional outcomes and hemorrhagic complications between low BP and standard management arms^9^. Multiple observational studies have shown varying results^10,11^. Because of the lack of any robust data, many guidelines either do not comment on absolute BP targets in the management of patients after successful reperfusion^12^, or they recommend the same BP targets suggested for post-intravenous thrombolysis care, i.e., ≤ 185/110 mm Hg^13^.

Biologically, leptomeningeal collateral (LMC) perfusion after LVO depends on the pressure gradient across these collateral channels; thus, augmentation of blood flow through LMCs using systemic BP modification may only work before recanalization is achieved. Moreover, elevated systemic BP may only be capable of increasing cerebral blood flow (CBF) when local cerebral autoregulation capacity is depleted, a condition that is likely influenced by the severity of baseline perfusion defect^14^. We, therefore, hypothesize that the baseline brain perfusion status likely influences the relationship between systemic BP and stroke outcome in patients with LVO and that such a relationship is present only before successful recanalization is achieved. To this end, we analyzed 388 anterior circulation LVO patients with post-EVT modified thrombolysis in cerebral infarction (mTICI) ≥ 2b who underwent intensive BP monitoring after arriving at the emergency department.

## Methods

### Ethical statement

Informed consent from patients or the next of kin was obtained for the prospective stroke registry under the approval of the Seoul Nation University Bundang Hospital institutional review board (IRB#, B-1706-403-303). All experimental protocols and analysis plans were approved by the Seoul Nation University Bundang Hospital institutional review board (IRB#, B-2102-667-105). All human research was conducted according to the Declaration of Helsinki.

### Study population

This was an observational, single-center, retrospective analysis of prospectively acquired data. Between January 2016 and March 2020, 580 endovascular recanalization treatments for acute ischemic stroke patients were performed in the Cerebrovascular Center of the Seoul National University Bundang Hospital, which participates in a prospective multicenter stroke registry^15^. Among the patients who underwent EVT, 192 were excluded due to (1) posterior circulation (n = 90), (2) poor-quality baseline images (n = 16), (3) lack of perfusion studies before EVT (n = 41), and (4) unsuccessful recanalization with mTICI less than 2a (n = 45).

Acute stroke management was performed as per institutional protocol based on the current guidelines at the time of practice and at the discretion of the individual physicians^13,16^. In our center, general anesthesia is not recommended during EVT, and conscious sedation is selectively administered to some uncooperative patients as determined by the treating team. Successful recanalization was defined as post-EVT mTICI 2b or 3. The time of recanalization was defined as the earliest time when successful recanalization was observed.

### Blood pressure management and clinical data collection

When patients arrive at our stroke center with stroke-like symptoms, BP recordings at 5-min intervals using a portable patient monitor (Mediana M30, Mediana Co. Ltd., Korea) were recorded, starting from hospital arrival. During the EVT, noninvasive BP was recorded using a monitoring device (IntelliVue MX450, Koninklijke Philips N.V, Netherland) until the end of the procedure as part of usual care. After EVT, all patients were transferred to a stroke unit where BP is measured as per the guidelines, i.e., at least every hour until 24 h after the procedure^13^. The institutional protocol recommends as per the acute care guideline to maintain SBP < 185 mm Hg; the uses of BP-lowering intravenous medications were decided by an interventionalist or attending physician after considering neurological deficit, BP level, and degree of recanalization. All BP measurements were recorded in the institutional registry database along with the time of the measurements. The use of BP-lowering medication and their time were retrospectively collected.

Baseline clinical information and follow-up functional recovery data were retrieved from the prospective stroke registry^15^. Data on the 3-month mRS score were prospectively collected during a regular clinic visit or through a structured telephone interview conducted by a trained research nurse.

### Image analysis

Follow-up images were routinely acquired 3–5 days after the procedure or at the time of neurologic deterioration to evaluate hemorrhagic complications and final infarct volume. Magnetic resonance imaging is the recommended imaging modality, but CT scans were accepted based on clinical circumstances.

Infarct proportion was measured as a ratio of the volume of final infarction over the volume of baseline T_max_ ≥ 6 s. The baseline T_max_ ≥ 6 s map from CT or MR perfusion images was reconstructed using Olea Sphere 3.0 (Olea Medical, La Ciotat, France). The perfusion map was exported to Analyze 14 (AnalyzeDirect, Inc., KS, USA) and co-registered to the follow-up diffusion-weighted images (DWI). Artifacts found on the T_max_ map were manually removed. Only lesions with high signal intensity on the DWI and an apparent diffusion coefficient less than 600 μm^2^/s within the region of T_max_ ≥ 6 s were counted as the final infarction. Infarct proportion was quantitated by a research technician with supervision by a vascular neurologist.

### Definitions of BP indices and imaging parameters

We summarized systolic BP (SBP) measurements with the following indices: SBP_dropmax_, the maximum decrease between two consecutive SBP measurements; SBP_incmax_, the maximum increase between two consecutive SBP measurements; SBP_mean_, the average of SBP measurements; SBP_sd_, the standard deviation of SBP measurements; and SBP_cv_, the coefficient of variation of SBP measurements.

$${\text{SBP}}_{\text{dropmax}} = \text{max(}{X}_{i} - {X}_{i+1}) \text{if (}{X}_{i} > {X}_{i+1})$$

$${\text{SBP}}_{\text{incmax}} = \text{max(}{X}_{i+1} - {X}_{i}) \text{if (}{X}_{i} < {X}_{i+1})$$

$${\text{SBP}}_{\text{mean}} = \frac{1}{N}\sum {X}_{i}$$

$${\text{SBP}}_{\text{sd}} = \sqrt{\frac{\sum {({x}_{i} - \overline{x })}^{2}}{(N - 1)}}$$

$${\text{SBP}}_{{{\text{cv}}}} ~ = ~{\text{SBP}}_{{{\text{sd}}}} /{\text{SBP}}_{{{\text{mean}}}}$$

Additionally, the range (SBP_max_–SBP_min_), the successive variation (SBP_sv_), and the average real variability (SBP_arv_) were analyzed against the infarct proportion and presented as supplemental data.

The hypoperfusion intensity ratio (HIR) was defined as the volume of tissue with T_max_ > 10 s divided by the volume of tissue with T_max_ > 6 s on the baseline CT or MR perfusion images^17^. Adequate and poor perfusions were defined taking HIR 0.5 as a cut-off point. The post-EVT mTICI grade was initially rated during acute care and verified post hoc by a neurointerventionalist (SHB). Alberta Stroke Programme Early Computed Tomography Score (ASPECTS) was evaluated on the first images, using computed tomography (CT) in 235 (60.6%) and diffusion-weighted image (DWI) in 153 (39.4%). ASPECTS were comparable between CT and DWI (median [interquartile range]; 9 ^7,9^] versus 8 [^6–10^]; *P*-for-difference, 0.36). Hemorrhagic transformation was evaluated on follow-up imaging using the Heidelberg Bleeding Classification (HBC)^18^. We defined significant hemorrhage as HBC class 2 or HBC class 3, i.e., PH2 hemorrhage or intracerebral hemorrhage outside the infarcted brain tissue or intracranial-extracerebral hemorrhage.

### Statistical analysis

Baseline characteristics were summarized and compared using the chi-squared test for categorical variables and an independent *t-test* for interval variables. Due to the right-skewed distribution of final infarct proportion, multivariable gamma regression models were used to test the effect of BP indices on this variable. Infarct proportion was designated as the primary outcome variable. Binary logistic regression models were used when the outcome was significant hemorrhage, while ordinal logistic regression models were used when the outcome was the overall distribution of mRS scores at three months. Multivariable models were adjusted for covariates with clinical significance or a bivariate *P* value < 0.10, including age, the time last known well to arrival, baseline NIHSS score, ASPECTS, occlusion location, hypertension, procedural sedation, and intravenous BP-lowering medication. Significance levels were set at *P* < 0.05, with all statistical tests being two-tailed. The *P*-values of multiplicative interaction terms were considered to have marginal significance when *P*-value was between ≥ 0.05 and < 0.10^19^. All tests were considered exploratory and hypothesis-generating; therefore, no adjustment was made for multiple tests^20^. All statistical analyses were performed using R, version 4.1.0 (R Foundation for Statistical Computing).

## Results

### Study population

A total of 388 anterior circulation LVO patients ≤ 24 h from their last known well (LKW) with baseline perfusion images were selected for the current analysis. The average age of the study population was 70.6 ± 12.7 years, and 39.7% of participants were female. The median baseline National Institutes of Health Stroke Scale (NIHSS) score was 14 [interquartile range (IQR), 8–18], and the median time from LKW to arrival was 3.3 h [IQR, 1.1–8.8]. Forty-nine percent (n = 188) of cases involved M1 occlusion at baseline, and intravenous thrombolysis was given in 31% (n = 122) of cases. The mean SBP at the initial presentation was 153 ± 52 mm Hg. No patient received general anesthesia during the EVT procedure, and 29% (n = 113) had conscious sedation. Patients with adequate baseline perfusion, i.e., baseline HIR < 0.5, were more likely to be female with atherosclerotic stroke and to have a lower baseline NIHSS score and longer last known well (LKW) to arrival interval. The adequate perfusion group had a relatively low infarction proportion but showed comparable significant hemorrhage and functional recovery to the poor baseline perfusion group. The five selected BP indices were not different according to the baseline perfusion subgroup (Table 1).

**Table 1:** Overall characteristics of included patients by baseline perfusion status.

Variables	All patients (n = 388)	Baseline HIR < 0.5 (n = 166)	Baseline HIR ≥ 0.5 (n = 219)	*P*-for-difference
Age	70.6 ± 12.7	70.2 ± 12.1	71.2 ± 12.5	0.421
Male sex	238 (61.3%)	86 (51.8%)	152 (69.4%)	0.001
Pre-stroke dependency (mRS ≥ 1)	129 (33.2%)	54 (32.5%)	75 (34.2%)	0.807
Baseline NIHSS score	14.0 [8.0–18.0]	11.0 [7.0–16.0]	15.0 [10.0–19.0]	< 0.001
LKW to arrival	3.3 [1.1–8.8]	4.7 [1.5–12.8]	2.4 [1.0–6.8]	< 0.001
Stroke mechanism				< 0.001
Large artery atherosclerosis	104 (26.8%)	60 (36.1%)	43 (19.6%)	
Cardioembolic	185 (47.7%)	59 (35.5%)	125 (57.1%)	
Other determined etiology	30 (7.7%)	14 (8.4%)	15 (6.8%)	
Undetermined etiology	69 (17.8%)	33 (19.9%)	36 (16.4%)	
Occlusion location				0.208
Extracranial ICA	42 (10.8%)	18 (10.8%)	24 (11.0%)	
Intracranial ICA	72 (18.6%)	22 (13.3%)	49 (22.4%)	
M1	188 (48.5%)	85 (51.2%)	102 (46.6%)	
M2 or distal	81 (20.9%)	38 (22.9%)	42 (19.2%)	
ACA	5 (1.3%)	3 (1.8%)	2 (0.9%)	
ASPECTS	8 [6–9]	9 [7–10]	8 [6–9]	0.002
Intravenous thrombolysis	122 (31.4%)	41 (24.7%)	80 (36.5%)	0.018
LKW to groin puncture (hours)	4.8 [2.5–11.0]	7.5 [3.1–15.7]	3.8 [2.1–8.9]	< 0.01
Hypertension	276 (71.1%)	114 (68.7%)	161 (73.5%)	0.354
Diabetes	133 (34.3%)	72 (43.4%)	61 (27.9%)	0.002
Dyslipidemia	134 (34.5%)	69 (41.6%)	65 (29.7%)	0.021
Smoking	137 (35.3%)	51 (30.7%)	86 (39.3%)	0.104
Atrial fibrillation	175 (45.1%)	61 (36.7%)	114 (52.1%)	0.004
Baseline SBP	153.4 ± 51.5	157.1 ± 71.5	150.6 ± 28.5	0.267
Baseline DBP	81.8 ± 50.0	84.9 ± 73.2	79.5 ± 19.0	0.355
Conscious sedation*	113 (29.1%)	35 (21.1%)	78 (35.6%)	0.003
Parenteral BP-lowering medication	39 (10.1%)	19 (11.4%)	19 (8.7%)	0.465
SBP indices				
SBPdropmax	54.3 ± 23.3	53.0 ± 19.1	55.6 ± 26.1	0.246
SBPincmax	50.3 ± 23.9	49.8 ± 20.4	50.9 ± 26.3	0.655
SBPcv	13.0 ± 4.2	13.0 ± 3.7	13.0 ± 4.5	0.915
SBPsd	17.2 ± 5.9	17.4 ± 5.6	17.2 ± 6.1	0.735
SBPmean	132.1 ± 13.2	133.2 ± 14.1	131.3 ± 12.4	0.173
Stroke outcomes				
Infarct proportion within Tmax > 6 s (%)	13.4 ± 17.1	10.6 ± 15.8	15.8 ± 17.8	0.01
	6.2 [1.6–20.1]	4.6 [1.6–12.3]	8.2 [1.9–25.8]	
Significant hemorrhage	47 (12.2%)	18 (10.9%)	29 (13.3%)	0.582
mRS 0–2 at three months	210 (54.5%)	90 (54.5%)	118 (54.4%)	0.999

*ASPECTS* Alberta Stroke Programme Early Computed Tomography Score, *HIR* hypoperfusion intensity ratio, *mRS* modified Rankin Scale, *NIHSS* National Institutes of Health Stroke Scale, *LKW* last known well, *ICA* internal carotid artery, *M1* M1 segment of a middle cerebral artery, *M2* M2 segment of a middle cerebral artery, *ACA* anterior cerebral artery, *SBP* systolic blood pressure, *DBP* diastolic blood pressure, *BP* blood pressure, *cv* coefficient of variation, *sd* standard deviation.

*No cases with general anesthesia.

### Intensive BP monitoring

**Figure 1:**
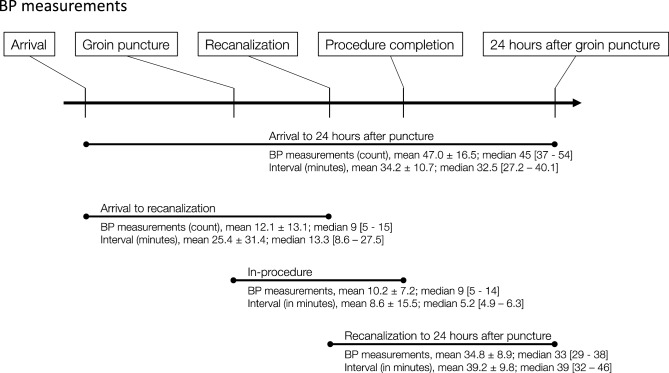
Intensive BP monitoring and the BP measurement profile.

All study subjects had intensive BP monitoring through a portable BP monitor or an installed monitoring device. The mean count (± standard deviation) of BP measurements was 47 ± 16.5 between arrival and 24 h after groin puncture, and the measurement interval was a median of 33 min [IQR, 27.2–40.1]. The median interval of BP monitoring during the EVT procedure was 5.2 min [IQR, 4.9–6.3]. The median counts of BP measurements were 9 [IQR, 5–15] in the pre-recanalization period and 33 [IQR, 29–38] in the post-recanalization period (Fig. 1).

The average SBP_mean_ and SBP_dropmax_ of the included patients were 148 ± 19.5 mm Hg and 34.2 ± 23.2 mm Hg in the pre-recanalization period and 128 ± 12.3 mm Hg and 49.0 ± 23.3 mm Hg in the post-recanalization period, respectively. Details on the SBP profiles from intensive BP monitoring during the peri-EVT periods are provided in Table 2.

**Table 2:** SBP profile from intensive BP monitoring during the peri-EVT periods.

		Arrival to recanalization	Recanalization to 24 h after EVT
SBPdropmax	Mean ± sd	34.2 ± 23.2	49.0 ± 23.3
	Median [IQR]	28 [17–45]	43 [33–60]
SBPincmax	Mean ± sd	30.5 ± 20.5	45.5 ± 22.5
	Median [IQR]	27 [16–43]	40 [30–54]
SBPmean	Mean ± sd	148 ± 19.5	128 ± 12.3
	Median [IQR]	148 [133–161]	128 [120–136]
SBPsd	Mean ± sd	14.3 ± 8.23	14.6 ± 4.9
	Median [IQR]	13.0 [8.5–17.8]	13.8 [11.1–17.2]
SBPcv	Mean ± sd	9.7 ± 5.4	11.5 ± 3.8
	Median [IQR]	8.9 [6.1–11.9]	10.7 [8.8–13.2]

**Figure 2:**
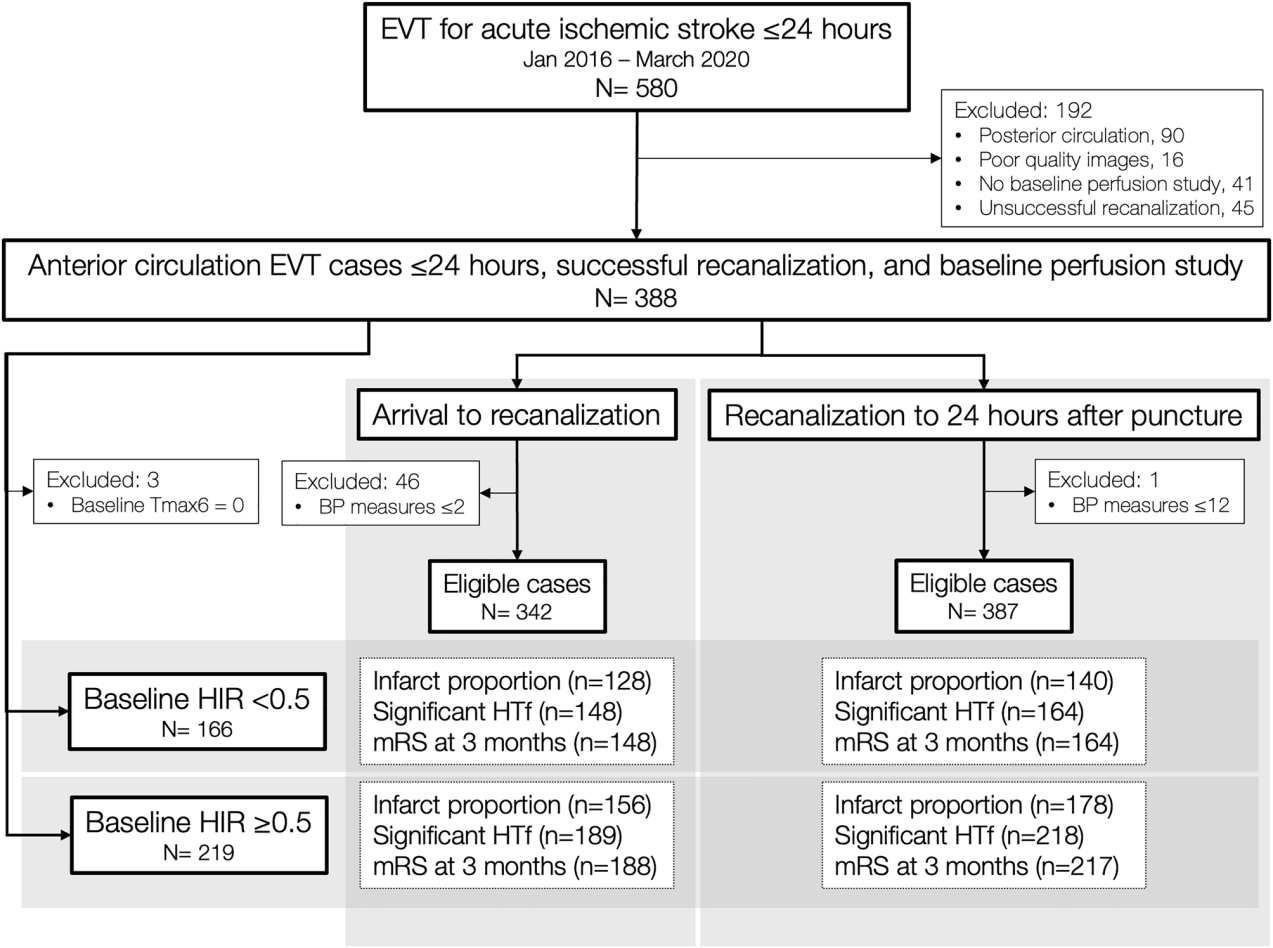
Study profile and case selection for multivariable models.

Among the 388 study subjects, we further selected cases with ≥ 3 BP measurements in the pre-recanalization modeling (n = 342) and ≥ 13 values in the post-recanalization modeling (n = 387) to reliably calculate the SBP indices (Fig. 2).

### BP indices and stroke outcomes by baseline perfusion and recanalization status

In EVT patients with successful recanalization, the association between SBP indices and stroke outcome varied according to the dichotomized baseline HIR and the pre- versus post-recanalization period (Table 3).

**Table 3:** Associations between BP indices and stroke outcomes by baseline perfusion status.

	Infarct proportion within Tmax > 6 s		Significant hemorrhage		Higher mRS score at 3 months	
	Arrival to recanalization	Recanalization to 24 h	Arrival to recanalization	Recanalization to 24 h	Arrival to recanalization	Recanalization to 24 h
LVO patients with adequate baseline perfusion (HIR < 0.5)						
SBPdropmax per 10 mm Hg	1.22 ± 0.48 (< 0.01)	2.04 ± 0.35 (< 0.01)	0.92 [0.68–1.23]	1.05 [0.75–1.47]	1.14 [0.99–1.32]	1.10 [0.93–1.30]
SBPincmax per 10 mm Hg	1.12 ± 0.33 (< 0.01)	2.07 ± 0.46 (< 0.01)	0.94 [0.72–1.23]	1.14 [0.85–1.52]	1.18 [1.03–1.36]	1.18 [1.01–1.38]
SBPcv	0.61 ± 0.15 (< 0.01)	− 0.04 ± 0.23 (0.84)	0.96 [0.82–1.12]	1.04 [0.87–1.23]	1.12 [1.03–1.21]	1.03 [0.94–1.13]
SBPsd	0.66 ± 0.08 (< 0.01)	0.04 ± 0.21 (0.85)	0.96 [0.87–1.06]	1.01 [0.88–1.14]	1.05 [1.004–1.10]	1.05 [0.98–1.12]
SBPmean per 10 mm Hg	0.71 ± 0.37 (0.053)	0.94 ± 0.42 (0.03)	0.84 [0.61–1.17]	0.81 [0.52–1.24]	0.89 [0.76–1.05]	1.22 [0.99–1.52]
LVO patients with poor baseline perfusion (HIR ≥ 0.5)						
SBPdropmax per 10 mm Hg	− 0.41 ± 0.37 (0.27)	0.08 ± 0.13 (0.54)	0.99 [0.82–1.21]	1.00 [0.85–1.17]	1.06 [0.95–1.17]	1.02 [0.92–1.13]
SBPincmax per 10 mm Hg	0.36 ± 0.48 (0.46)	0.08 ± 0.14 (0.57)	1.11 [0.89–1.38]	0.99 [0.83–1.17]	1.09 [0.94–1.26]	1.01 [0.90–1.12]
SBPcv	0.12 ± 0.12 (0.33)	0.07 ± 0.10 (0.52)	0.99 [0.91–1.07]	1.04 [0.94–1.15]	1.04 [0.99–1.09]	1.00 [0.94–1.07]
SBPsd	0.07 ± 0.09 (0.44)	0.05 ± 0.10 (0.61)	1.00 [0.95–1.06]	1.05 [0.97–1.14]	1.02 [0.99–1.05]	1.00 [0.95–1.05]
SBPmean per 10 mm Hg	− 0.45 ± 0. 30 (0.13)	− 0.28 ± 0.53 (0.60)	1.18 [0.93–1.51]	1.22 [0.81–1.83]	0.88 [0.76–1.03]	0.98 [0.78–1.23]

Multivariable models adjusted for age, the time last known well to arrival, baseline NIHSS score, ASPECTS, occlusion locations, history of hypertension, conscious sedation, and use of intravenous BP-lowering medications.

Infarct proportion within Tmax6: parameter estimate ± standard error (*P* values) from multivariable gamma regression models.

Significant hemorrhage: odds ratio [95% confidence interval] from multivariable binary logistic regression models.

Higher mRS score at 3 months: common odds ratio [95% confidence interval] from multivariable ordinal logistic regression models.

All five SBP indices analyzed in patients with adequate baseline perfusion, i.e., HIR < 0.5, showed significant association with infarct proportion in the pre-recanalization period (Table 3, upper panel). There was a 1.22% ± 0.48 increase in infarct proportion with every 10 mm Hg increase in SBP_dropmax_ (*P* < 0.01). Similarly, a positive correlation with infarct proportion was noted for SBP_incmax_, SBP_cv,_ and SBP_sd_. In the post-recanalization period, SBP_dropmax_, SBP_incmax_, and SBP_mean_ showed significant association with greater infarct proportion, but the significance was lost with SBP_cv_ and SBP_sd_. Likewise, a higher mRS score at three months, representing worse functional recovery, was associated with SBP_incmax_, SBP_cv,_ and SBP_sd_ in the pre-recanalization period and with SBP_incmax_ in the post-recanalization period. There was no correlation of significant hemorrhage with any of the SBP indices in the pre- and post-recanalization periods in the adequate perfusion subgroup.

These associations were non-existent in acute stroke patients with poor baseline perfusion status, i.e., HIR ≥ 0.5 (Table 3, lower panel). The infarct proportion was not associated with any of the five SBP indices regardless of recanalization periods. No SBP indices correlated with either a higher mRS score or significant hemorrhage in the poor perfusion subgroup. Unadjusted effect estimates are presented in Supplemental Table 1.

**Figure 3:**
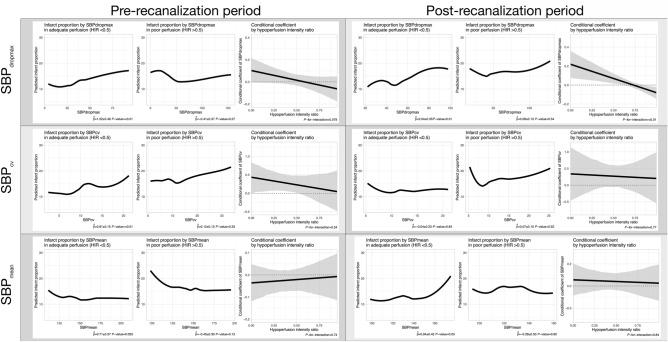
Estimated infarct proportions from the adequate and poor perfusion subgroups as well as conditional coefficients by the pre- and postrecanalization periods from SBP_dropmax_, SBP_cv_, and SBP_mean_. Each was selected on the premise of representing rapid fluctuation, global variability, and the overall level of SBP. Each panel contains the estimated infarct proportions within the Tmax > 6 s volume in the adequate perfusion (baseline HIR < 0.5) and poor perfusion (baseline HIR ≥ 0.5) subgroups and conditional coefficients of SBP indices by the HIR. SBP_dropmax_ and SBP_cv_ (the first and second row) of the adequate perfusion subgroup showed significant associations with the predicted infarct proportion in the pre-recanalization period (in the left-hand panels). However, their associations diminished in the poor perfusion subgroups (in the middle panels), and the conditional coefficient slopes of these indices were marginally significant only in SBP_dropmax_ (*P*-for-interaction, 0.079 and 0.34) by the level of baseline HIR (the third graph of each panel). In the postrecanalization period (the right-hand panels), the SBP_dropmax_ showed a positive correlation with infarct proportion only in the HIR < 0.5 group, and its conditional coefficient was significant (*P*-for-interaction, < 0.01). However, SBP_cv_ was not associated with infarct proportion in the post-recanalization period. Although SBP_mean_ (in the bottom row) showed a positive correlation with infarct proportion in post-recanalization periods of the adequate perfusion group (estimate ± standard error, 0.94 ± 0.42; *P*-value, 0.03), their coefficients were not modified by the level of baseline HIR (*P*-for-interaction, 0.74 for pre-recanalization and 0.84 for post-recanalization).

We further plotted the predicted infarct proportion from SBP_dropmax_, SBP_cv,_ and SBP_mean_ by the baseline perfusion and recanalization statuses and the conditional coefficient by the HIR range (Fig. 3). The rapid fluctuation of BP (SBP_dropmax_) showed marginal significance in the conditional coefficient in the pre-recanalization period (*P*-for-interaction, 0.079) and significance in the post-recanalization period (*P*-for-interaction, < 0.01) by the level of HIR. However, the associations between the global variability (SBPcv) or the overall level (SBPmean) and infarct proportion were not modified by the level of HIR in the peri-EVT periods (*P*-for-interactions, 0.34 and 0.77 for SBPcv and 0.74 and 0.84 for SBPmean).

We examined the relationship between additional SBP parameters, such as SBP_range_, SBP_sv_, and SBP_arv_, and infarct proportions (see Supplemental Table 3). Only SBP_range_ was significantly associated with infarct proportion in the post-recanalization period among the three parameters. We also examined the effect of BP-lowering medication during the acute 24 h on the infarct proportion, which was unaffected by SBP parameters regardless of baseline perfusion status (see Supplemental Table 4).

## Discussion

Pre- and post-recanalization, BP profiles have distinct effects on EVT outcomes. Pre-recanalization, SBP_dropmax_ and SBP_cv_ are associated with infarct proportion in subgroups with adequate perfusion, but these associations diminish in subgroups with inadequate perfusion. Only SBP_dropmax_ correlates with infarct proportion in HIR < 0.5 groups following recanalization. SBP_dropmax_ has a greater effect on groups with low baseline perfusion.

These results show that levels and changes of SBP before the recanalization had a significant effect on infarct proportion and functional recovery in patients with adequate perfusion distal to the LVO. The contribution of SBP indices on the mRS score was mitigated in the post-reperfusion period of the adequate perfusion group. No such relationship was noted in patients with poor baseline perfusion regardless of reperfusion status. There was no noticeable relationship between SBP indices and post-EVT hemorrhagic transformation in any models. The contribution of systemic BP to ischemic injury may differ by the baseline perfusion status, but not all BP indices were able to demonstrate the relationship. SBP_dropmax_ and the infarct proportion, among the selected SBP indices and stroke outcomes, were able to show the relationship between BP and ischemic injury.

As regional perfusion pressure decreases after LVO, the pial artery dilates to provide maximal blood flow within its capacity^21^. Misery LMC perfusion maintains the viability of the ischemic penumbra. The cerebral autoregulation capacity is likely already depleted in this region, and systemic perfusion pressure will be the only driving force of regional CBF^22^. Thus, BP fluctuations may contribute to changes in cerebral perfusion in the ischemic brain, especially before recanalization is achieved. In our study, the association between the SBP indices and stroke outcomes was not noted in the poor baseline perfusion group. This discrepancy may be due to the fact that either (1) widespread occlusion of capillaries and irreversible infarctions are already established^23^, or (2) these patients may have severely depressed LMC perfusion, unable even to cause any change in CBF from the fluctuation of systemic BP^24,25^. Patients with adequate baseline cerebral perfusion, with fully recruited LMCs alongside depleted autoregulatory function, may accommodate changes in systemic BP by changing CBF.

SBP levels and changes had no association with follow-up hemorrhage regardless of baseline perfusion status or recanalization period in our study. The reasoning that BP contributes to intracerebral hemorrhage is based on the idea that hydrostatic pressure causes the rupture of fragile arterioles or microaneurysms^26^. On the other hand, hemorrhagic transformation after EVT may be caused by ischemic injury to the cerebral vascular beds and parenchymal inflammation, resulting in increased permeability of the arteriolar or capillary wall and the extravasation of blood components into the brain tissue^27^. Thus, it may be inferred that hemorrhagic complications after recanalization treatment may be attributed not to BP changes but to recanalization itself and the microvascular ischemic injury that accumulated before recanalization.

Successful recanalization in LVO patients induces changes in cerebral hemodynamics. As forward flow is restored, the role of collateral flow through the LMC channel is reduced, and physiological levels of CBF are reinstated. Thus, the relationship between systemic BP and regional CBF may differ by the reperfusion status. Our study documented that the contributions from rapid fluctuation, i.e., SBP_dropmax_ and SBP_incmax_, or from the overall level, i.e., SBP_mean_, on the infarct proportion persist after recanalization. In contrast, the effect of global variability of BP, i.e., SBP_sd_ and SBP_cv_, on ischemic injury became negligible after recanalization.

Theoretically, systolic and diastolic BP measurements are generated with every cardiac contraction; BP can be measured repeatedly and changes according to various internal and external conditions. It is crucial to choose the most appropriate BP summary index for clinical or research purposes^10^. Mean BP, which reflects the steady and stable BP over a certain period, is probably the most widely used index, but it does not capture minute-by-minute fluctuations. It cannot be used to establish a causal relationship with an event during a measurement period^5^. Global BP variability has been summarized using the standard deviation, coefficient of variation, and variation independent of the mean, which together successfully demonstrate the long-term variation for stroke survivors^28^. However, their role in acute management is relatively limited in that the variability values vary depending on the BP measurement density, and the BP measurement interval is not incorporated in calculating them. The authors developed BP_dropmax_ and BP_incmax_, which are intuitive and easy to calculate and are applicable to acute stroke management^29^. These indices may quantify rapid fluctuations over a very short period. In the current study, these SBP indices behaved differently according to baseline perfusion and recanalization status. Thus, each of these BP indices may also have a specific role in understanding the effect of systemic BP on outcomes in patients with acute ischemic stroke.

Early infarct growth may be a function of time from onset, ischemic severity, i.e., baseline LMC perfusion, and tissue vulnerability^30^. It can be speculated that ischemic injury rapidly develops in a case with poor LMCs after LVO, and the only effective treatment strategy is to expedite recanalization. Augmentation of CBF with therapeutically elevated BP would work only when the local cerebral autoregulation capacity is depleted, and adequate LMC channels are present. In contrast, in the presence of moderate LMCs, tissue viability mainly depends on systemic perfusion, and clinicians should be sensitive to even minute changes in blood pressure. At the same time, in patients with excellent or good collaterals, one can expect a higher tolerance to modest BP fluctuations. This conjecture needs to be validated in the future.

BP levels and changes during the peri-EVT periods were found to aggravate the ischemic injury, especially in the pre-recanalization periods of the adequate baseline perfusion subgroup. However, their magnitudes of coefficient estimates were small, which may explain weak associations from BP indices with functional recovery three months after stroke.

In this study, we acknowledge that our current analysis does not capture the entire extent of blood pressure variability and its potential impact on patient outcomes. While our approach focused on the SBPdropmax, we recognize that incorporating additional methods, such as the delta (SBPmax–SBPmin), the average variability between measurements, or moving averages, could provide a more complete understanding of variability (see Supplemental Table 3). In addition, it is crucial to consider the U-shaped phenomenon associated with extremely high and low blood pressures, as it has been shown to affect outcomes in both hemorrhagic and ischemic infarcts. Although our data analysis does not delve into these factors, we believe that future research should investigate them in order to better comprehend the role of blood pressure variability in patient outcomes and potentially enhance the clinical management of such cases.

As the precise association between blood pressure profiles and stroke outcomes remains elusive, we acknowledge in this study that blood pressure medications play a role in the management of acute stroke patients. Despite the fact that our analysis did not prove the effects of blood pressure medications on variability (see Supplemental Table 4), we recognize that drug-modified blood pressure is a given condition for the BP profile of acute stroke patients. Future research should concentrate on the effect of blood pressure medications on patient outcomes and blood pressure variability in order to obtain a better understanding of the optimal blood pressure management for acute stroke patients.

A few points need further clarification. First, this is a retrospective study from a single center with all of the shortcomings associated with the design, resulting in a possible selection bias. However, we collected all the BP data according to an institutional protocol and selected the study subjects through prespecified steps to minimize selection bias. Second, we analyzed various outcome measures and multiple SBP indices, and multiple comparisons were inevitable in our analysis scheme. Inflation of type I error was possible, but we accepted a conventional level of significance considering the exploratory nature of our study^20^. Third, pre-and post-recanalization SBPs are inherently not independent due to repeated measurements. Fourth, we investigated SBP indices for increasing clinical practicality and reducing the BP data dimension. Fifth, we did not incorporate procedural information which could influence treatment outcomes. Sixth, anterior circulation LVO patients without perfusion images were excluded from analyses (see Supplemental Table 2 for the baseline characteristics of excluded 41 cases). Lastly, LVO patients with unsuccessful recanalization were excluded from this study to preclude the ongoing ischemic injury after the EVT.

## Conclusion

We documented that the contribution of the SBP profile to stroke outcomes may depend on the baseline perfusion and the recanalization status in anterior circulation LVO patients with successful recanalization. SBP during the pre-recanalization period is equally important and is a determinant of outcomes, and the contribution may be affected by the baseline perfusion status. Our study suggests that a more sophisticated BP management strategy during the acute stroke period is required and needs to reflect various conditions, such as baseline perfusion, recanalization, and cerebral autoregulation capacity.

### Supplementary Information

Supplementary Tables.

## Data Availability

Data supporting the study results will be provided as a reasonable request from qualified researchers directed to the corresponding author.
