# Supplementary material for: Association between blood pressure and endovascular treatment outcomes differs by baseline perfusion and reperfusion status
Source: Sci Rep. 2023 Aug 23;13:13776. doi: 10.1038/s41598-023-40572-0 (PMC10447432; doi:10.1038/s41598-023-40572-0)
Supplement: Supplementary file 1 — Supplementary Tables. [file 41598_2023_40572_MOESM1_ESM.docx]

Supplemental data

**Association between Blood Pressure and Endovascular Treatment Outcomes Differs by Baseline Perfusion and Reperfusion Status**

Beom Joon Kim, Nihista Singh, Hyeran Kim, et al.

Supplemental Table 1. Unadjusted effect estimates

| LVO patients with adequate baseline perfusion (HIR <0.5) | | | | | | | |
| --- | --- | --- | --- | --- | --- | --- | --- |
|  |  | Infarct proportion within Tmax >6 seconds | | Significant hemorrhage | | Higher mRS score at 3 months | |
|  |  | Arrival to recanalization | Recanalization to 24 hours | Arrival to recanalization | Recanalization to 24 hours | Arrival to recanalization | Recanalization to 24 hours |
|  | SBPdropmax per 10 mm Hg | 1.13 ± 0.70 (0.11) | -0.22 ± 0.70 (0.75) | 0.99 [0.78 - 1.27] | 1.02 [0.79 - 1.33] | 1.19 [1.04 - 1.37] | 1.19 [1.03 - 1.37] |
|  | SBPincmax per 10 mm Hg | 0.45 ± 0.59 (0.45) | 1.25 ± 0.75 (0.10) | 1.07 [0.85 - 1.34] | 1.12 [0.88 - 1.42] | 1.18 [1.03 - 1.35] | 1.25 [1.08 - 1.44] |
|  | SBPcv | 0.67 ± 0.31 (0.03) | 0.11 ± 0.39 (0.78) | 1.04 [0.92 - 1.16] | 1.05 [0.91 - 1.20] | 1.13 [1.05 - 1.21] | 1.11 [1.02 - 1.20] |
|  | SBPsd | 0.32 ± 0.20 (0.11) | 0.11 ± 0.29 (0.72) | 1.01 [0.94 - 1.08] | 1.02 [0.91 - 1.13] | 1.06 [1.02 - 1.10] | 1.09 [1.03 - 1.16] |
|  | SBPmean per 10 mm Hg | 1.13 ± 0.70 (0.11) | -0.22 ± 0.70 (0.75) | 0.90 [0.69 - 1.17] | 0.81 [0.56 - 1.18] | 0.95 [0.82 - 1.10] | 1.16 [0.94 - 1.42] |
| LVO patients with poor baseline perfusion (HIR ≥0.5) | | | | | | | |
|  |  | Infarct proportion within Tmax >6 seconds | | Significant hemorrhage | | Higher mRS score at 3 months | |
|  |  | Arrival to recanalization | Recanalization to 24 hours | Arrival to recanalization | Recanalization to 24 hours | Arrival to recanalization | Recanalization to 24 hours |
|  | SBPdropmax per 10 mm Hg | -0.23 ± 0.63 (0.72) | 0.50 ± 0.71 (0.48) | 1.01 [0.85 - 1.19] | 1.02 [0.88 - 1.18] | 1.11 [1.01 - 1.23] | 1.12 [1.02 - 1.23] |
|  | SBPincmax per 10 mm Hg | -0.19 ± 0.85 (0.82) | 0.47 ± 0.78 (0.55) | 1.11 [0.91 - 1.37] | 1.00 [0.85 - 1.17] | 1.09 [0.95 - 1.25] | 1.15 [1.04 - 1.27] |
|  | SBPcv | 0.09 ± 0.31 (0.77) | 0.06 ± 0.41 (0.89) | 0.99 [0.93 - 1.07] | 1.07 [0.98 - 1.17] | 1.06 [1.02 - 1.20] | 1.07 [1.01 - 1.13] |
|  | SBPsd | 0.07 ± 0.21 (0.72) | 0.14 ± 0.33 (0.67) | 1.00 [0.96 - 1.05] | 1.06 [0.99 - 1.14] | 1.04 [1.01 - 1.07] | 1.06 [1.01 - 1.11] |
|  | SBPmean per 10 mm Hg | -0.23 ± 0.64 (0.72) | 0.50 ± 0.71 (0.48) | 1.14 [0.92 - 1.42] | 1.03 [0.73 - 1.45] | 0.98 [0.86 - 1.12] | 1.12 [0.91 - 1.38] |

Supplemental Table 2. Baseline characteristics of 41 excluded patients due to lack of baseline perfusion images

|  |  |  |
| --- | --- | --- |
| Variables | Included patients (n=388) | Excluded patients  (n=41) |
| Age | 70.6 ± 12.7 | 75.4 ± 10.2 |
| Male sex | 238 (61.3%) | 20 (48.8%) |
| Pre-stroke dependency (mRS ≥1) | 129 (33.2%) | 19 (46.3%) |
| Baseline NIHSS score | 14.0 [8.0-18.0] | 12 [8-16] |
| LKW to arrival | 3.3 [1.1-8.8] | 4.3 [1.8-14.6] |
| Stroke mechanism |  |  |
| Large artery atherosclerosis | 104 (26.8%) | 13 (31.7%) |
| Cardioembolic | 185 (47.7%) | 18 (43.9%) |
| Other determined etiology | 30 (7.7%) | 4 (9.8%) |
| Undetermined etiology | 69 (17.8%) | 6 (14.6) |
| Occlusion location |  |  |
| Extracranial ICA | 42 (10.8%) | 7 (17.1%) |
| Intracranial ICA | 72 (18.6%) | 5 (12.2%) |
| M1 | 188 (48.5%) | 16 (39.0%) |
| M2 or distal | 81 (20.9%) | 13 (31.7%) |
| ACA | 5 (1.3%) | 0 |
| Intravenous thrombolysis | 122 (31.4%) | 14 (34.1%) |
| ASPECTS | 8 [6-9] | 9 [7-9] |
| Hypertension | 276 (71.1%) | 33 (80.5%) |
| Diabetes | 133 (34.3%) | 11 (26.8%) |
| Dyslipidemia | 134 (34.5%) | 17 (41.5%) |
| Smoking | 137 (35.3%) | 14 (34.1%) |
| Atrial fibrillation | 175 (45.1%) | 19 (46.3%) |
| Baseline SBP | 153.4 ± 51.5 | 154.8 ± 31.3 |
| Baseline DBP | 81.8 ± 50.0 | 80.2 ± 19.8 |
| Conscious sedation * | 113 (29.1%) | 22 (53.7%) |
| Parenteral BP-lowering medication | 39 (10.1%) | 7 (17.1%) |
| SBP indices |  |  |
| SBPdropmax | 54.3 ± 23.3 | 52.6 ± 23.1 |
| SBPincmax | 50.3 ± 23.9 | 49.5 ± 20.6 |
| SBPcv | 13.0 ± 4.2 | 12.8 ± 4.9 |
| SBPsd | 17.2 ± 5.9 | 17.6 ± 6.9 |
| SBPmean | 132.1 ± 13.2 | 135.5 ± 16.4 |
| Stroke outcomes |  |  |
| Infarct proportion within Tmax >6 sec (%) | 13.4 ± 17.1 | - |
|  | 6.2 [1.6-20.1] |  |
| Significant hemorrhage | 47 (12.2%) | 7 (17.1%) |
| mRS 0-2 at three months | 210 (54.5%) | 10 (24.4%) |
| * No cases with general anesthesia | |  |

Supplemental Table 3. Associations between additional BP indices representing BP variability and the infarct proportion

| BP parameter | HIR | Arrival to recanalization | | Recanalization to 24 hours | |
| --- | --- | --- | --- | --- | --- |
|  |  | Infarct proportion | P-for interaction | Infarct proportion | P-for interaction |
| SBP range per 10 mm Hg | <0.5 | 1.16 ± 0.33 (<0.01) | 0.25 | 2.37 ± 0.40 (<0.01) | <0.01 |
|  | ≥0.5 | -0.29 ± 0.34 (0.40) |  | 0.01 ± 0.13 (0.49) |  |
| SBPsv per 10 mm Hg | <0.5 | 4.38 ± 1.06 (<0.01) | 0.55 | 5.40 ± 1.08 (<0.01) | 0.32 |
|  | ≥0.5 | 1.02 ± 0.96 (0.29) |  | 0.53 ± 0.75 (0.48) |  |
| SBParv per 10 mm Hf | <0.5 | 6.97 ± 1.31 (<0.01) | 0.69 | 7.04 ± 1.49 (<0.01) | 0.16 |
|  | ≥0.5 | 1.04 ± 1.13 (0.35) |  | 1.31 ± 1.70 (0.44) |  |

Multivariable models adjusted for age, the time last known well to arrival, baseline NIHSS score, ASPECTS, occlusion locations, history of hypertension, conscious sedation, and use of intravenous BP-lowering medications.

Infarct proportion within Tmax6: parameter estimate ± standard error (P values) from multivariable gamma regression models

P-for-interactions were from the interaction between HIR and BP parameters.

Supplemental Table 4. Associations between BP-lowering medication and the infarct proportion in the main models

| LVO patients with adequate baseline perfusion (HIR <0.5) | | | |
| --- | --- | --- | --- |
|  |  | Infarct proportion within Tmax >6 seconds | |
|  | BP parameter models | Arrival to recanalization | Recanalization to 24 hours |
|  | SBPdropmax per 10 mm Hg | 1.12 ± 3.40 (0.74) | 0.95 ± 1.43 (0.51) |
|  | SBPincmax per 10 mm Hg | 0.29 ± 3.36 (0.93) | -0.63 ± 2.47 (0.80) |
|  | SBPcv | 1.37 ± 1.90 (0.24) | 5.97 ± 3.18 (0.06) |
|  | SBPsd | -0.20 ± 2.12 (0.92) | 0.54 ± 4.57 (0.91) |
|  | SBPmean per 10 mm Hg | 2.01 ± 3.89 (0.61) | 4.38 ± 4.18 (0.30) |
| LVO patients with poor baseline perfusion (HIR ≥0.5) | | | |
|  |  | Infarct proportion within Tmax >6 seconds | |
|  | BP parameter models | Arrival to recanalization | Recanalization to 24 hours |
|  | SBPdropmax per 10 mm Hg | -0.48 ± 2.06 (0.82) | -1.08 ± 1.52 (0.48) |
|  | SBPincmax per 10 mm Hg | -0.50 ± 1.42 (0.72) | -1.40 ± 1.59 (0.38) |
|  | SBPcv | -1.62 ± 1.78 (0.36) | -1.10 ± 1.36 (0.42) |
|  | SBPsd | -1.32 ± 1.76 (0.45) | -0.60 ± 1.41 (0.67) |
|  | SBPmean per 10 mm Hg | -0.63 ± 1.32 (0.63) | -0.35 ± 1.20 (0.77) |

Multivariable models adjusted for age, the time last known well to arrival, baseline NIHSS score, ASPECTS, occlusion locations, history of hypertension, conscious sedation, and BP parameters from each model.

Infarct proportion within Tmax6: parameter estimate ± standard error (P values) from multivariable gamma regression models
